# Ligand Screening Systems for Human Glucose Transporters as Tools in Drug Discovery

**DOI:** 10.3389/fchem.2018.00183

**Published:** 2018-05-25

**Authors:** Sina Schmidl, Cristina V. Iancu, Jun-yong Choe, Mislav Oreb

**Affiliations:** ^1^Institute of Molecular Biosciences, Goethe University Frankfurt, Frankfurt am Main, Germany; ^2^Department of Biochemistry and Molecular Biology, Rosalind Franklin University of Medicine and Science, North Chicago, IL, United States

**Keywords:** glucose transport, sugar transport inhibitors, screening system, sugar transport assays, drug discovery, hxt^0^ strain

## Abstract

Hexoses are the major source of energy and carbon skeletons for biosynthetic processes in all kingdoms of life. Their cellular uptake is mediated by specialized transporters, including glucose transporters (GLUT, SLC2 gene family). Malfunction or altered expression pattern of GLUTs in humans is associated with several widespread diseases including cancer, diabetes and severe metabolic disorders. Their high relevance in the medical area makes these transporters valuable drug targets and potential biomarkers. Nevertheless, the lack of a suitable high-throughput screening system has impeded the determination of compounds that would enable specific manipulation of GLUTs so far. Availability of structural data on several GLUTs enabled *in silico* ligand screening, though limited by the fact that only two major conformations of the transporters can be tested. Recently, convenient high-throughput microbial and cell-free screening systems have been developed. These remarkable achievements set the foundation for further and detailed elucidation of the molecular mechanisms of glucose transport and will also lead to great progress in the discovery of GLUT effectors as therapeutic agents. In this mini-review, we focus on recent efforts to identify potential GLUT-targeting drugs, based on a combination of structural biology and different assay systems.

## Introduction

In human cell membranes, glucose transporter family members (GLUT, gene family SLC2) facilitate the diffusion of glucose and related monosaccharides along the concentration gradient. The 14 GLUTs are grouped according to phylogenetic homology into 3 classes: class I with GLUT1-4 (all transport glucose, GLUT2 also transports fructose), class II with GLUT5, 7, 9, and 11 (all transport fructose and glucose except for GLUT5—a fructose-only transporter; GLUT9 also transports uric acid), and class III with GLUT6, 8, 10, 12, and 13 (all transport glucose, except for GLUT13, which is a myo-inositol/proton symporter) (Thorens and Mueckler, 2010; Mueckler and Thorens, 2013). Other GLUT substrates are galactose, mannose, glucosamine, and dehydroascorbic acid. GLUTs differ in transport capacity, substrate affinity and specificity, and tissue distribution; the latter reflects local physiological needs. Alterations in the function, localization or expression of GLUTs are associated with Mendelian disorders (Santer et al., 1997; Seidner et al., 1998), cancer (Thorens and Mueckler, 2010; Barron et al., 2016), diabetes (Elsas and Longo, 1992), obesity (Song and Wolfe, 2007), gout (George and Keenan, 2013), non-alcoholic fatty liver disease (Douard and Ferraris, 2013), and renal disease (Kawamura et al., 2011). Thus GLUTs are important subjects for medical research and show great potential as drug targets for the treatment of a number of these diseases for instance in cancer therapy. It is known that cancer cells show an increased expression of glucose transporters to meet their need for higher energy demand due to uncontrolled proliferation (Warburg, 1956; Cairns et al., 2011). A higher expression rate of several GLUTs has already been identified in various kinds of tumors (Szablewski, 2013). Most prominently, higher expression rates of GLUT1 have been found in most cancer tissues (Godoy et al., 2006) and studies indicate that this overexpression is an early event in the course of the disease (Rudlowski et al., 2003; Macheda et al., 2005). Various studies also related abnormal expression of other transporters, including GLUT4, GLUT6, GLUT7, GLUT8, GLUT11, and GLUT12, with the fast proliferation of cancer cells (Rogers et al., 2002; Godoy et al., 2006; McBrayer et al., 2012); GLUT5 was found in breast cancer tissue but was absent in normal breast tissue (Zamora-León et al., 1996). Metabolites which specifically modify the activity of certain GLUT isoforms would therefore be very valuable for cancer therapy which is furthermore encouraged by studies showing that cancer cells die faster than normal cells under glucose-limiting conditions (Liu et al., 2010).

Diabetes mellitus type 2 is another prominent example of a GLUT-related disease whereas GLUT4 is considered a key player in the pathogenesis of this disease. This transporter is predominantly expressed in adipose tissue, heart, and skeletal muscle and is stored in small vesicles in the cytoplasm until insulin triggers its translocation to the plasma membrane, where it mediates glucose uptake (Hajiaghaalipour et al., 2015). Diabetic type 2 cells show diminished expression of GLUT4 as well as impaired trafficking to the plasma membrane (Patel et al., 2006). Furthermore, a proper anchoring of GLUT2 at the surface of β-cells seems to be crucial for the physiological glucose-uptake in these cells which is in turn required for normal glucose-stimulated insulin secretion (Ohtsubo et al., 2005). Reduced stability of GLUT2 in the plasma membrane disrupts insulin secretion and therefore favors the development of type 2 diabetes (Ohtsubo et al., 2005). Substrates with the ability to modulate altered functions of GLUTs involved in the pathogenesis of diabetes might contribute to the therapy and diminish symptoms of diabetes type 2 patients.

Given the complex role that GLUTs play in different diseases the discovery of GLUT-selective is highly desirable. Recent advances in three-dimensional structure determination of GLUTs and their homologs (Sun et al., 2012; Iancu et al., 2013; Deng et al., 2014; Nomura et al., 2015) finally make structure-based drug design possible, as exemplified by two HIV integrase inhibitors Raltegravir and Elvitegravir (Williamson et al., 2012). In particular, *in silico* ligand screening studies have uncovered GLUT-specific inhibitors for the first time. In this mini-review article, we will summarize the current efforts to identify potential GLUT-targeting drugs, based on a combination of structural biology and different assay systems.

## Structure-based discovery of compounds targeting GLUTs

GLUTs belong to the sugar porter family of the Major Facilitator Superfamily (MFS) proteins (Saier et al., 1999; www.tcdb.org), one of the largest and most ubiquitous protein families. As other MFS proteins, GLUTs have 12 transmembrane helices organized into two 6-helices domains (the N- and C-halves); a central polar cavity formed between the N- and C-domains contains the substrate binding site. GLUTs have an alternating access transport mechanism whereby the substrate cavity presents in turn to either the lumen (outward-facing conformation) or cytoplasm (inward-facing conformation). Crystal structures of GLUTs and their homologs have captured outward- and inward-facing conformations, in different ligation states (apo, with substrate or inhibitors), with the substrate cavity open (open conformation) to or partially shielded (occluded conformation) from solvent (Sun et al., 2012; Iancu et al., 2013; Deng et al., 2014; Nomura et al., 2015; Kapoor et al., 2016; see Table 1). Comparison of the crystal structures of GLUT1 inward-open conformation and GLUT3 outward-facing conformations (outward-occluded and –open), suggest that the alternating access mechanism involves a rigid-body rotation of the N-terminal half relative to the C-terminal half and rearrangements in the substrate interactions with residues mostly from the C-terminal domain (Deng et al., 2015). Ligand docking studies of substrate and inhibitors to different conformations of GLUT1, based on crystal structures of GLUT1, GLUT3 and the bacterial homolog XylE, show conformation-dependent variation in the number and location of the ligand binding sites: several potential glucose binding sites (three for the outward-open conformation, two for the outward-occluded conformation and one each for the inward-occluded and inward-open conformations) and, in the case of GLUT1 inhibitors, two maltose binding sites in the outward-facing conformation, and two sites for cytochalasin B in the outward-facing conformation (Lloyd et al., 2017). Obviously, structure-based ligand screening for GLUTs will need to employ all available conformations of a transporter.

**Table 1:** Crystal structures of GLUTs and their homologs.

**Protein**	**Source**	**Conformation**	**PDB ID**	**References**
Xylose/H^+^ symporter	*Escherichia coli*	Outward-occluded	4GBY	Sun et al., 2012
			4GBZ	
			4GC0	
		Inward-open	4JA4	Quistgaard et al., 2013
			4JA3	
		Inward-open	4QIQ	Wisedchaisri et al., 2014
Glucose/H^+^ symporter	*Staphylococcus epidermidis*	Inward-open	4LDS	Iancu et al., 2013
GLUT1	*Homo sapiens*	Inward-open	4PYP	Deng et al., 2014
		Inward-open	5EQI	Kapoor et al., 2016
			5EQG	
			5EGH	
GLUT3	*Homo sapiens*	Outward-occluded	4ZW9	Deng et al., 2015
		Outward-occluded	4ZWB	
		Outward-open	4ZWC	
		Outward-occluded	5C65	Pike et al., 2015
GLUT5	*Rattus*	Outward-facing	4YBQ	Nomura et al., 2015
	*Bos taurus*	Inward-open	4YB9	

Structure-based drug discovery relies on reliable 3D structures of a target protein, *in silico* ligand screening with libraries of small compounds, and assay systems to validate and characterize the ligand candidates. Subsequent rounds of chemical optimization, informed by structure-based design, may further increase the potency and specificity of the identified ligands (Sliwoski et al., 2014; Schreiber et al., 2015).

So far, *in silico* ligand screening has been reported for GLUT1, GLUT4, and GLUT5 (Mishra et al., 2015; George Thompson et al., 2016; Ung et al., 2016). This is a high-throughput ligand screening method in which millions of small compounds are assessed computationally for their ability to bind to a target structure (Colas et al., 2016). Table 2 lists GLUT inhibitors with IC_50_ under 20 μM uncovered through *in silico* ligand screening studies. Human GLUT crystal structures were unavailable at the time of the initial virtual screening, so structural models were based on the crystal structures of bacterial GLUT homologs or other MFS proteins (Table 2) and represented either the inward-facing conformation (GLUT4 and GLUT5) or the outward-facing conformation (GLUT1). The number of molecules in the screen library varied: ~550,000 (Fragment Now and NCI-2007) for GLUT1, ~6 million (Chemnavigator) for GLUT5, and ~ 10 million (ZINC) for GLUT4. The number of resulting ligand candidates purchased and checked for activity against GLUTs was 17, 19, and 175, respectively, for GLUT4, GLUT1, and GLUT5. The transport assay systems were: GLUT1-expressing CHO cells, GLUT4-expressing HEK293 cells or multiple myeloma cell lines, and GLUT5 proteoliposomes. The studies identified eight GLUT1 inhibitors (including compounds A and B in Table 2), two GLUT4 inhibitors (compound E is a structural derivative of compound C, Table 2) and one GLUT5 inhibitor. Inhibitor selectivity was not established for GLUT1, but was determined at different extents for GLUT4 and GLUT5 inhibitors. Thus, compounds C and D (Table 2), seemed selective for GLUT4, compared to GLUT1, which is impressive given the extensive amino acid sequence conservation in the substrate binding cavity between these class I GLUTs. Compound F, did not affect the glucose transport of GLUT1, 2, 3 or 4, or the fructose transport of GLUT2, in proteoliposomes, proving to be a GLUT5-specific inhibitor. Mutagenesis studies on GLUT1, 5 and the bacterial GLUT homolog GlcP_Se_ confirmed the predicted binding site of compound F in GLUT5 and identified His 387 of GLUT5 as a residue important in inhibitor selectivity. Nevertheless, whether compound F remains GLUT5-selective, compared to other class II GLUTs, in particular GLUT7, which has the equivalent of His 387, remains to be established. Subsequent chemical optimization has been done only for GLUT4 inhibitors so far. Based on compound E, Wei et al. performed SAR (structure–activity relationship) analysis and antagonist synthesis and found that compound E analogs decreased proliferation of the plasma cell malignancy multiple myeloma (Wei et al., 2017).

**Table 2:** Leading probes for GLUTs from *in silico* ligand screening.

	**Target protein**	**Template PDB ID**	**Screen library**	**Chemical IDs**	**Smiles**	**Synomyms**	**IC50 (μM)**	**References**
A	GLUT1 Outward	4CGO (XylE)	Fragment Now, NCI-2007	PubChem CID: 417049	C1=CC=C(C=C1)C (C2=NC3C(=NC= NC3=O)N2)O	8-[hydroxy(phenyl) methyl]-5,9-dihydropurin-6-one	0.45	1
B	GLUT1 Outward	4CGO (XylE)	Fragment Now, NCI-2007	ZINC 17003013, PubChem CID: 250016, CAS: 13617-04-4	c1c2c(c(=O)[nH] n1)Sc3cn[nH]c(= O)c3S2	[1,4]dithiino[2,3-d:5,6-d']dipyridazine-1,6-diol	11.8	1
C	GLUT4 Inward	1PW4 (GlpT), 1PV6 (LacY), 2GFP (EmrD)	ZINC	ZINC 14974263, ChemBridge: 59900452	COc1ccccc1CCC( =O)N(Cc2ccncc2) Cc3cccc(c3)OCCc 4ccc(cc4)F	N-{3-[2-(4-fluorophenyl)ethoxy]benzyl}-3-(2-methoxyphenyl) -N-(4-pyridinylmethyl)propanamide	18.9	2
D	GLUT4 Inward	1PW4 (GlpT), 1PV6 (LacY), 2GFP (EmrD)	ZINC	ZINC 11785066, ChemBridge: 27190707	C[NH+](Cc1cccc( c1)OC[C@@H]2C CCN(C2)C(=O)c3 c4c([nH]n3)CCC4 )Cc5ccc6c(c5)ccc n6	N-methyl-1-(6-quino linyl)-N-(3-{[1-(1,4,5,6-tetrahydrocyclopenta[c]pyrazol-3-ylcarbonyl)-3-piperidinyl]methoxy}benzyl)methanamine	10.8	2
E	GLUT4 Inward	1PW4 1PV6, 2GFP	ZINC	ZINC 12152508, ChemBridge: 55751832	COc1ccc(cc1)C(= O)N(Cc2ccncc2)C c3cccc(c3)OCCc4 ccc(cc4)F	N-[[3-[2-(4-fluorophenyl)ethoxy]phenyl]methyl]-4-methoxy-N-(4-pyridyl methyl)benzamide	6.67	2
F	GLUT5 Inward	4LDS (GlcP_Se_)	Chem Navigator	Structure_ID: 32283234, Enamine: Z31191163	[S](=O)(=O)(C)c1 cc(c(cc1)Nc2cc3c (cc2)OCO3)[N+]( =O)[O–]	N-[4-(methylsulfonyl)-2-nitrophenyl]-1,3-benzodioxol-5-amine, MSNBA	5.8	3
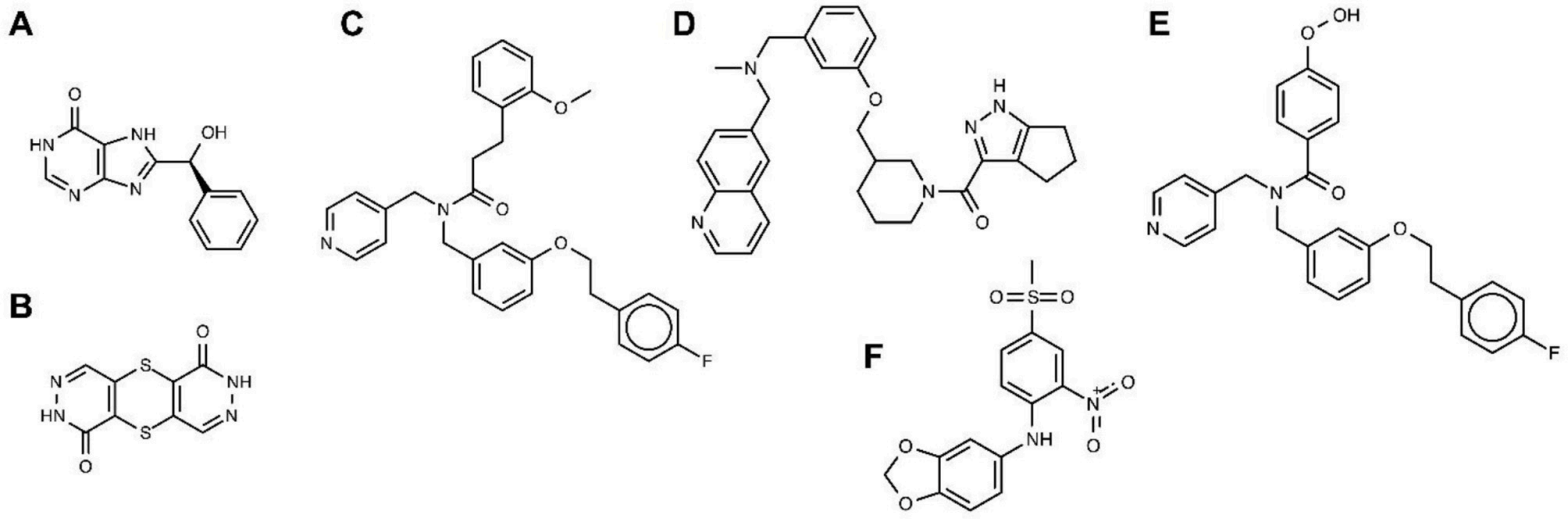								

*References: 1. Ung et al. (2016); 2. Mishra et al. (2015); 3. George Thompson et al. (2016)*.

All the described *in silico* studies are a promising start for drug discovery efforts targeting GLUTs. Now that crystal structures for several human GLUTs are available, *in silico* studies for all GLUTs are possible. Furthermore, for the same GLUTs, inhibitors for the outward-facing and inward-facing conformations can be identified. To further establish the selectivity of the new inhibitors, GLUT-specific assay systems are required.

## Assays and screening systems for GLUT activity

*In silico* approaches usually yield a large number of compounds that need to be evaluated for their effect on hexose transport by GLUTs to select the best candidates for possible (pre)clinical trials. Thereby, an ideal assay system should be quick and inexpensive, but at the same time it must preserve the transporter's properties, e.g., in terms of transport kinetics.

*In vitro* (cell-free) systems offer the advantage of a strictly defined composition, which minimizes the risk of non-controllable interferences as often encountered in a complex cellular context. Thereby, studies of membrane proteins require the simulation of their native lipid environment. Different approaches have been tested with purified GLUTs to fulfill this task. Kraft et al. (2015) succeeded in producing milligram amounts of rat GLUT4 in mammalian HEK293 cells and were able to reconstitute correctly folded protein into detergent micelles, amphipols, nanodiscs and proteoliposomes. The latter are suitable for transport assays by constituting a two-compartment (outside/inside) system (Saier et al., 1999; Geertsma et al., 2008). By allowing lateral diffusion and the generation of a membrane curvature, this system best mimics the native surroundings of GLUTs compared to other *in vitro* systems (Kraft et al., 2015). A noteworthy advantage of proteoliposomes is the fact that parameters like the lipid composition or the degree of membrane curvature can be varied systematically. For instance, Hresko et al. (2016) could show that presence of anionic phospholipids in the proteoliposomes stabilized reconstituted GLUT3 and GLUT4 while conical lipids enhanced the transport rate. Besides considerable advantages, the liposome reconstitution approach also bears some drawbacks. First, for membrane reconstitution, a sufficient amount of purified protein is necessary. A general instability of membrane proteins outside of their native lipid environment and the shortcomings of most purification methods, concerning the purity or the yield of the target protein, makes the heterologous expression and purification of structurally and functionally stable protein time-, labor- and cost-intensive (Geertsma et al., 2008; Kraft et al., 2015). Furthermore, many factors have to be taken into account and optimized for a successful membrane reconstitution, such as the application of a suitable (mild or harsh) detergent, its concentration as well as the protein-to-lipid ratio, protein-orientation and the choice for either synthetic lipids or lipid extracts (Geertsma et al., 2008) resulting in a complex handling. Nevertheless, progress has been made in the various fields of protein purification and stability (Kraft et al., 2015), improving the utility of proteoliposomes for transport assays. For instance, proteoliposomes were successfully used to assess the effect of inhibitors of GLUT1 (George Thompson et al., 2015) and GLUT5 (George Thompson et al., 2015, 2016).

As a complementary approach that avoids laborious protein purification and reconstitution procedures, different cell-based systems for assaying GLUTs have been employed.

Functional expression of membrane proteins in *Xenopus laevis* oocytes opened the gate for closer molecular characterization (Hediger et al., 1987). Early experiments on GLUT1-5 in this expression system already yielded valuable information about the kinetic properties, substrate selectivity and effective inhibitors of the transporters (Birnbaum, 1989; Gould and Lienhard, 1989; Keller et al., 1989; Kayano et al., 1990; Gould et al., 1991). The system was proven to be suitable for investigating GLUT functions, due to a low endogenous GLUT expression in frog oocytes (Gould and Lienhard, 1989). Additionally, the large size of these cells facilitates handling and allows their application for electrophysiological experiments (Long et al., 2018). However, not all GLUT isoforms integrate properly into the oocyte plasma membrane and calculating their abundancy in membrane is not trivial (Gould and Lienhard, 1989; Keller et al., 1989). Furthermore, *Xenopus laevis* oocytes might be too instable for the application in high-throughput screening assays (César-Razquin et al., 2015).

Investigations on GLUTs can also be performed by expression in human cell lines such as MCF-7 or Caco-2 cells as it has been shown for GLUT2 and GLUT5 (Mahraoui et al., 1994; Zamora-León et al., 1996; Lee et al., 2015). In these systems, parameters such as lipid composition, posttranslational modifications and protein trafficking are most likely identical to the native conditions, although some alterations in cultured cells are, at least principally, possible. However, mammalian cell lines endogenously express several GLUT isoforms with overlapping activity, making it difficult to establish unambiguously the GLUT member(s) targeted by a compound (Lee et al., 2015; Tripp et al., 2017).

More recently, efforts have concentrated on establishing a microbial system amenable to high-throughput screening of GLUT inhibitors. Due to the easy manipulation and short generation time, the yeast *Saccharomyces cerevisiae* provides a time-efficient, low-cost and versatile platform for this purpose (Tripp et al., 2017; Boles and Oreb, 2018). For exclusive uptake of hexoses via heterologously expressed GLUTs, all genes encoding endogenous transporters capable of hexose transport (*HXT1-17, GAL2* as well as the maltose transporter genes *AGT1, MPH2*, and *MPH3*) were deleted in the yeast strain background CEN.PK2-1C using the loxP-Cre recombinase system (Wieczorke et al., 1999). The resulting strain was named EBY.VW4000 and is unable to take up and grow on glucose or related hexoses as sole carbon source. The functional expression of human GLUTs in this hexose transporter deficient (hxt^0^) yeast strain restores its ability to grow on glucose or fructose enabling compound screening for the particular human GLUT via simple cell growth assays (Wieczorke et al., 2002). Even though cell growth is the simplest parameter to determine the functionality of the transporters or potency of the inhibitors, compound screening is not limited to this method. For instance, yeast cells can be conveniently used for uptake assays of radiolabeled sugars, which allows for the determination of kinetic parameters of the transporters, including inhibitor constants (Maier et al., 2002; Tripp et al., 2017; Boles and Oreb, 2018).

However, the functional expression of human GLUTs in yeast cells require additional modifications either within the transporter or in the genome of the yeast strain. Whereas wildtype GLUT1, GLUT4 and GLUT5 (Kasahara and Kasahara, 1996, 1997; Wieczorke et al., 2002; Tripp et al., 2017) were not active in the hxt^0^ strain, single point mutations in the transmembrane region 2 of GLUT1 and GLUT5 mediated their functional expression (Wieczorke et al., 2002; Tripp et al., 2017). Wild-type GLUT1 was active only in the hxt^0^ strain that additionally acquired the *fgy1* (for functional expression of GLUT1 in yeast) mutation (Wieczorke et al., 2002). The affected gene encodes the Efr3 protein (Wieczorke and Boles, personal communication) that was later described as a scaffold for recruiting the Stt4 phosphatidylinositol-4-kinase to the plasma membrane and therefore necessary for normal phosphatidylinositol-4-phosphate levels in this compartment (Wu et al., 2014). The functional expression of GLUT4 required, in addition to *fgy1*, the *fgy4* mutation, that was later found to affect the *ERG4* gene (Boles et al., 2004), which encodes an enzyme involved in the last step of ergosterol biosynthesis. These observations suggest that the lipid composition of yeast membranes interferes with the functionality of GLUTs. Nevertheless, GLUT1, GLUT4 (Wieczorke et al., 2002), and GLUT5 (Tripp et al., 2017) expressed in yeast exhibited transport kinetic parameters comparable to those determined in liposomes or human cell lines and were responsive to established inhibitors of these transporters. Therefore, hxt^0^ strains represent a convenient platform for screening approaches and characterization of human GLUTs in a high throughput manner. The discovery of specific effectors for one certain GLUT, which do not influence homologs of the same protein family, is challenging due to the high protein sequence similarity shared by the members of this family (George Thompson et al., 2015). Among existing screening systems, the microbial, high-throughput screening system is the most effective method to face this challenge. Its usage and expansion to other disease-relevant GLUTs will likely reveal new GLUT-specific effectors which might be of fundamental importance for clinical applications in the battle against widespread diseases like cancer or diabetes.

## Author contributions

All authors listed have made a substantial, direct and intellectual contribution to the work, and approved it for publication.

### Conflict of interest statement

The authors declare that the research was conducted in the absence of any commercial or financial relationships that could be construed as a potential conflict of interest.

## References

[B1] Barron C. C. Bilan P. J. Tsakiridis T. Tsiani E. (2016). Facilitative glucose transporters. Implications for cancer detection, prognosis and treatment. Metabolism 65, 124–139. 10.1016/j.metabol.2015.10.007 26773935

[B2] Birnbaum M. J. (1989). Identification of a novel gene encoding an insulin-responsive glucose transporter protein. Cell 57, 305–315. 10.1016/0092-8674(89)90968-9 2649253

[B3] Boles E. Dlugai S. Mueller G. Voss D. (2004). Use of Saccharomyces Cerevisiae ERG4 Mutants for the Expression of Glucose Transporters From Mammals. WO002004026907A3. Geneva: World Intellectual Property Organization.

[B4] Boles E. Oreb M. (2018). A growth-based screening system for hexose transporters in yeast. Methods Mol. Biol. 1713, 123–135. 10.1007/978-1-4939-7507-5_10 29218522

[B5] Cairns R. A. Harris I. S. Mak T. W. (2011). Regulation of cancer cell metabolism. Nat. Rev. Cancer 11, 85–95. 10.1038/nrc2981 21258394

[B6] César-Razquin A. Snijder B. Frappier-Brinton T. Isserlin R. Gyimesi G. Bai X.. (2015). A call for systematic research on solute carriers. Cell 162, 478–487. 10.1016/j.cell.2015.07.022 26232220

[B7] Colas C. Ung P. M.-U. Schlessinger A. (2016). SLC transporters. structure, function, and drug discovery. Med. Chem. Comm. 7, 1069–1081. 10.1039/C6MD00005C 27672436 PMC5034948

[B8] Deng D. Xu C. Sun P. Wu J. Yan C. Hu M.. (2014). Crystal structure of the human glucose transporter GLUT1. Nature 510, 121–125. 10.1038/nature13306 24847886

[B9] Deng D. Sun P. Yan C. Ke M. Jiang X. Xiong L.. (2015). Molecular basis of ligand recognition and transport by glucose transporters. Nature 526, 391–396. 10.1038/nature14655 26176916

[B10] Douard V. Ferraris R. P. (2013). The role of fructose transporters in diseases linked to excessive fructose intake. J. Physiol. (Lond). 591, 401–414. 10.1113/jphysiol.2011.215731 23129794 PMC3577529

[B11] Elsas L. J. Longo N. (1992). Glucose transporters. Annu. Rev. Med. 43, 377–393. 10.1146/annurev.me.43.020192.002113 1580597

[B12] Geertsma E. R. Nik Mahmood N. A. Schuurman-Wolters G. K. Poolman B. (2008). Membrane reconstitution of ABC transporters and assays of translocator function. Nat. Protoc. 3, 256–266. 10.1038/nprot.2007.519 18274528

[B13] George R. L. Keenan R. T. (2013). Genetics of hyperuricemia and gout. Implications for the present and future. Curr. Rheumatol. Rep. 15:309. 10.1007/s11926-012-0309-8 23307580

[B14] George Thompson A. M. Iancu C. V. Nguyen T. T. Kim D. Choe J. Y. (2015). Inhibition of human GLUT1 and GLUT5 by plant carbohydrate products; insights into transport specificity. Sci. Rep. 5:12804. 10.1038/srep12804 26306809 PMC4549712

[B15] George Thompson A. M. Ursu O. Babkin P. Iancu C. V. Whang A. Oprea T. I.. (2016). Discovery of a specific inhibitor of human GLUT5 by virtual screening and *in vitro* transport evaluation. Sci. Rep. 6:160. 10.1038/srep24240 27074918 PMC4831007

[B16] Gould G. W. Thomas H. M. Jess T. J. Bell G. I. (1991). Expression of human glucose transporters in *Xenopus* oocytes: kinetic characterization and substrate specificities of the erythrocyte, liver, and brain isoforms. Biochemistry 30, 5139–5145. 10.1021/bi00235a004 2036379

[B17] Gould G. W. Lienhard G. E. (1989). Expression of a functional glucose transporter in *Xenopus* oocytes. Biochemistry 28, 9447–9452. 10.1021/bi00450a030 2692709

[B18] Godoy A. Ulloa V. Rodríguez F. Reinicke K. Yañez A. J. GarcíaMde L.. (2006). Differential subcellular distribution of glucose transporters GLUT1-6 and GLUT9 in human cancer. ultrastructural localization of GLUT1 and GLUT5 in breast tumor tissues. J. Cell. Physiol. 207, 614–627. 10.1002/jcp.20606 16523487

[B19] Hajiaghaalipour F. Khalilpourfarshbafi M. Arya A. (2015). Modulation of glucose transporter protein by dietary flavonoids in type 2 diabetes mellitus. Int. J. Biol. Sci. 11, 508–524. 10.7150/ijbs.11241 25892959 PMC4400383

[B20] Hediger M. A. Coady M. J. Ikeda T. S. Wright E. M. (1987). Expression cloning and cDNA sequencing of the Na^+^/glucose co-transporter. Nature 330, 379–381. 10.1038/330379a0 2446136

[B21] Hresko R. C. Kraft T. E. Quigley A. Carpenter E. P. Hruz P. W. (2016). Mammalian glucose transporter activity is dependent upon anionic and conical phospholipids. J. Biol. Chem. 291, 17271–17282. 10.1074/jbc.M116.730168 27302065 PMC5016126

[B22] Iancu C. V. Zamoon J. Woo S. B. Aleshin A. Choe J. Y. (2013). Crystal structure of a glucose/H^+^ symporter and its mechanism of action. Proc. Natl. Acad. Sci. U.S.A. 110, 17862–17867. 10.1073/pnas.1311485110 24127585 PMC3816430

[B23] Kapoor K. Finer-Moore J. S. Pedersen B. P. Caboni L. Waight A. Hillig R. C.. (2016). Mechanism of inhibition of human glucose transporter GLUT1 is conserved between cytochalasin B and phenylalanine amides. Proc. Natl. Acad. Sci. U.S.A. 113, 4711–4716. 10.1073/pnas.1603735113 27078104 PMC4855560

[B24] Kasahara T. Kasahara M. (1996). Expression of the rat GLUT1 glucose transporter in the yeast *Saccharomyces cerevisiae*. Biochem. J. 315, 177–182. 10.1042/bj3150177 8670104 PMC1217168

[B25] Kasahara T. Kasahara M. (1997). Characterization of rat Glut4 glucose transporter expressed in the yeast *Saccharomyces cerevisiae*: comparison with Glut1 glucose transporter. Biochim. Biophys. Acta 1324, 111–119. 10.1016/S0005-2736(96)00217-9 9059504

[B26] Kawamura Y. Matsuo H. Chiba T. Nagamori S. Nakayama A. Inoue H.. (2011). Pathogenic GLUT9 mutations causing renal hypouricemia type 2 (RHUC2). Nucleos. Nucleot. Nucl. 30, 1105–1111. 10.1080/15257770.2011.623685 22132964

[B27] Kayano T. Burant C. F. Fukumoto H. Gould G. W. Fan Y. S. Eddy R. L.. (1990). Human facilitative glucose transporters. J. Biol. Chem. 265, 13276–13282. 1695905

[B28] Keller K. Strube M. Mueckler M. (1989). Functional expression of the human HepG2 and rat adipocyte glucose transporters in *Xenopus* Oocytes. J. Biol. Chem. 264, 18884–18889. 2553725

[B29] Kraft T. E. Hresko R. C. Hruz P. W. (2015). Expression, purification, and functional characterization of the insulin-responsive facilitative glucose transporter GLUT4. Prot. Sci. 24, 2008–2019. 10.1002/pro.2812 26402434 PMC4815238

[B30] Lee Y. Lim Y. Kwon O. (2015). Selected phytochemicals and culinary plant extracts inhibit fructose uptake in Caco-2 Cells. Molecules 20, 17393–17404. 10.3390/molecules200917393 26393568 PMC6331785

[B31] Liu Y. Zhang W. Cao Y. Liu Y. Bergmeier S. Chen X. (2010). Small compound inhibitors of basal glucose transport inhibit cell proliferation and induce apoptosis in cancer cells via glucose-deprivation-like mechanisms. Cancer Lett. 298, 176–185. 10.1016/j.canlet.2010.07.002 20678861

[B32] Lloyd K. P. Ojelabi O. A. De Zutter J. K. Carruthers A. (2017). Reconciling contradictory findings: Glucose transporter 1 (GLUT1) functions as an oligomer of allosteric, alternating access transporters. J. Biol. Chem. 292, 21035–21046. 10.1074/jbc.M117.815589 29066623 PMC5743077

[B33] Long W. O'Neill D. Cheeseman C. I. (2018). GLUT characterization using frog *Xenopus laevis* Oocytes. Methods Mol. Biol. 1713, 45–55. 10.1007/978-1-4939-7507-5_4 29218516

[B34] Macheda M. L. Rogers S. Best J. D. (2005). Molecular and cellular regulation of glucose transporter (GLUT) proteins in cancer. J. Cell. Physiol. 202, 654–662. 10.1002/jcp.20166 15389572

[B35] Mahraoui L. Takeda J. Mesonero J. Chantret I. Dussaulx E. Bell G. I.. (1994). Regulation of expression of the human fructose transporter (GLUT5) by cyclic AMP. Biochem. J. 301, 169–175. 10.1042/bj3010169 8037665 PMC1137157

[B36] Maier A. Völker B. Boles E. Fuhrmann G. F. (2002). Characterisation of glucose transport in *Saccharomyces cerevisiae* with plasma membrane vesicles (countertransport) and intact cells (initial uptake) with single Hxt1, Hxt2, Hxt3, Hxt4, Hxt6, Hxt7 or Gal2 transporters. FEMS Yeast Res. 2, 539–550. 10.1111/j.1567-1364.2002.tb00121.x 12702270

[B37] McBrayer S. K. Cheng J. C. Singhal S. Krett N. L. Rosen S. T. Shanmugam M. (2012). Multiple myeloma exhibits novel dependence on GLUT4, GLUT8, and GLUT11. Implications for glucose transporter-directed therapy. Blood 119, 4686–4697. 10.1182/blood-2011-09-377846 22452979 PMC3367873

[B38] Mishra R. K. Wei C. Hresko R. C. Bajpai R. Heitmeier M. Matulis S. M.. (2015). *In silico* modeling-based identification of glucose transporter 4 (GLUT4)-selective inhibitors for cancer therapy. J. Biol. Chem. 290, 14441–14453. 10.1074/jbc.M114.628826 25847249 PMC4505511

[B39] Mueckler M. Thorens B. (2013). The SLC2 (GLUT) family of membrane transporters. Mol. Aspects Med. 34, 121–138. 10.1016/j.mam.2012.07.001 23506862 PMC4104978

[B40] Nomura N. Verdon G. Kang H. J. Shimamura T. Nomura Y. Sonoda Y.. (2015). Structure and mechanism of the mammalian fructose transporter GLUT5. Nature 526, 397–401. 10.1038/nature14909 26416735 PMC4618315

[B41] Ohtsubo K. Takamatsu S. Minowa M. T. Yoshida A. Takeuchi M. Marth J. D. (2005). Dietary and genetic control of glucose transporter 2 glycosylation promotes insulin secretion in suppressing diabetes. Cell 123, 1307–1321. 10.1016/j.cell.2005.09.041 16377570

[B42] Patel N. Huang C. Klip A. (2006). Cellular location of insulin-triggered signals and implications for glucose uptake. Pflugers Arch. 451, 499–510. 10.1007/s00424-005-1475-6 16284741

[B43] Pike A. C. W. Quigley A. Chu A. Tessitore A. Xia X. Mukhopadhyay S. (2015). Structure of the Human Glucose Transporter GLUT3/SLC2A3. Available online at: http://www.rcsb.org/structure/5C65

[B44] Quistgaard E. M. Löw C. Moberg P. Trésaugues L. Nordlund P. (2013). Structural basis for substrate transport in the GLUT-homology family of monosaccharide transporters. Nat. Struct. Mol. Biol. 20, 766–768. 10.1038/nsmb.2569 23624861

[B45] Rogers S. Macheda M. L. Docherty S. E. Carty M. D. Henderson M. A. Soeller W. C.. (2002). Identification of a novel glucose transporter-like protein-GLUT-12. Am. J. Physiol. 282, 733–738. 10.1152/ajpendo.2002.282.3.E733 11832379

[B46] Rudlowski C. Becker A. J. Schroder W. Rath W. Büttner R. Moser M. (2003). GLUT1 messenger RNA and protein induction relates to the malignant transformation of cervical cancer. Am. J. Clin. Pathol. 120, 691–698. 10.1309/4KYNQM5862JW2GD7 14608894

[B47] Saier M. H. Beatty J. T. Goffeau A. Harley K. T. Heijne W. H. Huang S. C.. (1999). The major facilitator superfamily. J. Mol. Microbiol. Biotechnol. 1, 257–279. 10943556

[B48] Santer R. Schneppenheim R. Dombrowski A. Götze H. Steinmann B. Schaub J. (1997). Mutations in *GLUT2*, the gene for the liver-type glucose transporter, in patients with Fanconi-Bickel syndrome. Nat. Genet. 17, 324-−326. 10.1038/ng1197-324 9354798

[B49] Schreiber S. L. Kotz J. D. Li M. Aubé J. Austin C. P. Reed J. C.. (2015). Advancing biological understanding and therapeutics discovery with small-molecule probes. Cell 161, 1252–1265. 10.1016/j.cell.2015.05.023 26046436 PMC4564295

[B50] Seidner G. Alvarez M. G. Yeh J. I. O'Driscoll K. R. Klepper J. Stump T. S.. (1998). GLUT-1 deficiency syndrome caused by haploinsufficiency of the blood-brain barrier hexose carrier. Nat. Genet. 18, 188–191. 10.1038/ng0298-188 9462754

[B51] Sliwoski G. Kothiwale S. Meiler J. Lowe E. W. (2014). Computational methods in drug discovery. Pharmacol. Rev. 66, 334–395. 10.1124/pr.112.007336 24381236 PMC3880464

[B52] Song D. H. Wolfe M. M. (2007). Glucose-dependent insulinotropic polypeptide and its role in obesity. Curr. Opin. Endocrinol. 14, 46–51. 10.1097/MED.0b013e328011aa88 17940419

[B53] Sun L. Zeng X. Yan C. Sun X. Gong X. Rao Y.. (2012). Crystal structure of a bacterial homologue of glucose transporters GLUT1-4. Nature 490, 361–366. 10.1038/nature11524 23075985

[B54] Szablewski L. (2013). Expression of glucose transporters in cancers. Biochim. Biophys. Acta 1835, 164–169. 10.1016/j.bbcan.2012.12.004 23266512

[B55] Thorens B. Mueckler M. (2010). Glucose transporters in the 21st Century. Am. J. Physiol. Endocrinol. 298, E141–E145. 10.1152/ajpendo.00712.2009 20009031 PMC2822486

[B56] Tripp J. Essl C. Iancu C. V. Boles E. Choe J. Y. Oreb M. (2017). Establishing a yeast-based screening system for discovery of human GLUT5 inhibitors and activators. Sci. Re. 7:124. 10.1038/s41598-017-06262-4 28740135 PMC5524692

[B57] Ung P. M. Song W. Cheng L. Zhao X. Hu H. Chen L.. (2016). Inhibitor discovery for the human GLUT1 from homology modeling and virtual screening. ACS Chem. Biol. 11, 1908–1916. 10.1021/acschembio.6b00304 27128978 PMC5356226

[B58] Warburg O. (1956). On the origin of cancer cells. Science 3191, 309–314. 10.1126/science.123.3191.309 13298683

[B59] Wei C. Bajpai R. Sharma H. Heitmeier M. Jain A. D. Matulis S. M.. (2017). Development of GLUT4-selective antagonists for multiple myeloma therapy. Eur. J. Med. Chem. 139, 573–586. 10.1016/j.ejmech.2017.08.029 28837922 PMC5603412

[B60] Wieczorke R. Dlugai S. Krampe S. Boles E. (2002). Characterisation of mammalian GLUT glucose transporters in a heterologous yeast Expression system. Cell. Physiol. Biochem. 13, 123–134. 10.1159/000071863 12876383

[B61] Wieczorke R. Krampe S. Weierstall T. Freidel K. Hollenberg C. P. Boles E. (1999). Concurrent knock-out of at least 20 transporter genes is required to block uptake of hexoses in *Saccharomyces cerevisiae*. FEBS Lett. 464, 123–128. 10.1016/S0014-5793(99)01698-1 10618490

[B62] Williamson E. A. Damiani L. Leitao A. Hu C. Hathaway H. Oprea T.. (2012). Targeting the transposase domain of the DNA repair component Metnase to enhance chemotherapy. Cancer Res. 72, 6200–6208. 10.1158/0008-5472.CAN-12-0313 23090115 PMC3972061

[B63] Wisedchaisri G. Park M. S. Iadanza M. G. Zheng H. Gonen T. (2014). Proton-coupled sugar transport in the prototypical major facilitator superfamily protein XylE. Nat. Comm. 5:4521. 10.1038/ncomms5521 25088546 PMC4137407

[B64] Wu X. Chi R. J. Baskin J. M. Lucast L. Burd C. G. De Camilli P.. (2014). Structural insights into assembly and regulation of the plasma membrane phosphatidylinositol 4-Kinase Complex. Dev. Cell 28, 19–29. 10.1016/j.devcel.2013.11.012 24360784 PMC4349574

[B65] Zamora-León S. P. Golde D. W. Concha II. Rivas C. I. Delgado-López F. Baselga J.. (1996). Expression of the fructose transporter GLUT5 in human breast cancer. Proc. Natl. Acad. Sci. U.S.A. 93, 1847–1852. 10.1073/pnas.93.5.1847 8700847 PMC39870

